# Effects of epidural anesthesia in pheochromocytoma and paraganglioma surgeries: A protocol for systematic review and meta-analysis

**DOI:** 10.1097/MD.0000000000031768

**Published:** 2022-11-25

**Authors:** Min Yang, Chao Kang, Shuai Zhu

**Affiliations:** a Department of Anesthesiology, Northwest Women’s and Children’s Hospital, Xi’an, China; b Department of Anesthesiology, The First Affiliated Hospital of Xi’an Jiaotong University, Xi’an, China.

**Keywords:** epidural anesthesia, general anesthesia, hypertensive crisis, hypotension, paraganglioma, pheochromocytoma

## Abstract

Anesthetic management is a great challenge during the surgical resection of pheochromocytomas and paragangliomas (PPGLs) due to potential hemodynamic fluctuations and/or postoperative complications. Although combined epidural-general anesthesia is commonly used in PPGLs surgeries, there is still no consensus whether combined epidural-general anesthesia was superior than general anesthesia alone for these populations. For the first time, we conducted this systematic review and meta-analysis to summarize the effects of combined epidural-general anesthesia versus general anesthesia alone on hemodynamic fluctuations as well as postoperative complications in patients undergone PPGLs surgeries.

**Methods:**

This systematic review and meta-analysis was performed according to the preferred reporting items for systematic reviews and meta-analyses statement. The primary outcome were hemodynamic fluctuations, including intraoperative hypotension, postoperative hypotension, and hypertensive crisis. Secondary outcome was the incidence of postoperative complications during hospital stay.

**Results:**

Finally, three retrospective cohort studies involving 347 patients met the inclusion criteria. A meta-analysis was not performed since outcomes from included studies were not available to be pooled. On the basis of the findings of non-randomized controlled trials (RCTs) literature, 2 studies suggested that combined epidural-general anesthesia was associated with intraoperative and postoperative hypotension, although one study reported that epidural anesthesia use reduced the incidence of postoperative complications in patients undergone surgical resection of PPGLs.

**Conclusions:**

Currently, no published RCTs have yet assessed clinically relevant outcomes with respect to the application of epidural anesthesia during PPGLs surgeries. Well-designed RCTs should nonetheless be encouraged to properly assess the efficacy and safety of epidural anesthesia for PPGLs surgeries.

## 1. Introduction

Pheochromocytomas and paragangliomas (PPGLs) are rare neuroendocrine tumors deriving from the chromaffin cells of the adrenal medulla and extra-adrenal neural crest tissues.^[1–3]^ The incidence of PPGLs is estimated up to 0.8 in 1,00,000 persons every year, with 80% to 85% pheochromocytomas and 15% to 20% paragangliomas cases.^[4,5]^ The common symptoms of PPGLs mainly include hypertension, palpitation, throbbing headache, and diaphoresis that induced by an excessive secretion of catecholamines.^[6]^ The diagnosis of PPGLs requires both evidence of excessive release of catecholamines and the anatomical localization of catecholamines-secreting tumor.^[7]^ The tumor-node-metastasis (TNM) system for PPGLs, based on the extent of the primary tumor (*T* stage), the extent of lymph node metastases (*N* stage), and the presence of distant metastases (*M* stage), has been introduced in the 8th Edition of the AJCC Cancer Staging Manual.^[8]^ Surgical resection, including open and laparoscopic approach, is the mainstay for treatment of PPGLs. Although laparoscopic resection is superior in terms of many aspects, open resection is more recommended for large or locally invasive pheochromocytomas, or multifocal tumors including metastases.^[9]^ Previous study indicated that surgical resection of the primary tumor could improve overall survival in patients with metastatic PPGLs.^[10]^ In addition to surgical resection, chemotherapy and radiopharmaceutical treatments also showed potential benefits for patients with PPGLs, such as the combined chemotherapy protocol of cyclophosphamide, vincristine, and dacarbazine, and radiopharmaceutical treatments using ^131^I-MIBG and ^131^I-Ultratrace Iobenguane.^[6]^

During PPGLs surgeries, anesthetic management is a great challenge due to dramatic hemodynamic fluctuations, such as hypotension, hypertensive crisis, and high occurrence risk of postoperative complications.^[5,11]^ Generally, general anesthesia is the standard anesthetic technique for the surgical resection of PPGLs.^[12,13]^ In recent years, epidural anesthesia gains superiority when used as a supplementary technique of general anesthesia in many aspects, including postoperative analgesia, reduction of opioids consumption, less cardiovascular and respiratory complications.^[14,15]^ Therefore, the combined epidural-general anesthesia has been frequently used in PPGLs surgeries.^[16–18]^ Increasing numbers of studies reported that epidural anesthesia may prevent the fluctuations in hormone levels^[19]^ and facilitate hemodynamic stability for patients undergoing PPGLs surgeries.^[20–23]^ However, epidural anesthesia was also implicated in intraoperative and/or postoperative hypotension due to decreased peripheral vascular tone,^[23,24]^ which might be worsen for PPGLs surgeries because of alpha-adrenoceptor blockade and abrupt reduction of circulating catecholamine levels.^[17,25]^ Epidural anesthesia was even reported to exacerbate intraoperative hypotension but not prevent hypertensive crisis during PPGLs surgery, which may be result from the fact that epidural block was not effective to suppress the catecholamine surge during tumor manipulation but exaggerate the sympathectomy and vasodilatory effects after tumor ligation.^[16]^ Several studies have compared the effects of combined epidural-general anesthesia with general anesthesia alone on hemodynamic instability and postoperative complications in patients undergone PPGLs surgeries,^[16–18]^ but the results remain controversial. It is thus worthwhile to conduct a systematic review and meta-analysis to summarize the efficacy and safety of epidural anesthesia in the surgical resection of PPGLs.

## 2. Materials and methods

### 2.1. Ethical statements

No ethical approval is required because this is a literature-based study. This systematic review and meta-analysis was conducted in agreement with the preferred reporting items for systematic reviews and meta-analyses guideline.^[26]^

### 2.2. Search strategy

PubMed, EMBASE, Cochrane Central Register of Controlled Trials (CENTRAL), and Web of science were systematically searched from the inception to June 3, 2021 with the following terms: “pheochromocytoma or paraganglioma,” “general anesthesia or general anesthesia” and “epidural.” The language was restricted to English. Additional studies were obtained by reviewing the references of relevant articles.

### 2.3. Inclusion and exclusion

Inclusion criteria: Study design: Randomized controlled trials (RCTs) or retrospective cohort studies; Population: adult patients undergoing surgical resection of PPGLs; Intervention: combined epidural-general anesthesia; Control: general anesthesia alone; and Primary outcomes: hemodynamic fluctuations, including intraoperative hypotension, postoperative hypotension, and hypertensive crisis; Secondary outcome: the incidence of postoperative complications during hospital stay. Reviews, conference abstracts, comments, and case reports were all excluded.

### 2.4. Study selection

Two authors independently screened the titles and abstracts of all the studies obtained from the search for the potentially relative studies. Then, the full-text of potentially eligible studies were retrieved and reviewed for study inclusion. Any disagreements were discussed with the third author.

### 2.5. Data extraction

Two authors independently extracted the following data from included studies: authors, publication year, study design, sample number, participants’ age, types of surgery, anesthetics of epidural anesthesia, intraoperative vasoactive medications, and primary outcomes. We contacted corresponding authors of included studies for missing or unreported data. Discrepancies were resolved by discussion with the third author.

### 2.6. Risk of bias assessment

Two authors independently assessed the risk of bias for each included study by the Robins-I tool.^[27]^ The assessment contains the following items: confounding, selection of participants, departure from intended interventions, missing data, measurement of outcomes, and selective reporting. The estimated risk of bias for each item was rated as “low,” “moderate,” “serious,” or “critical.” Any disagreements were discussed with the third author.

## 3. Results

**Figure 1.**
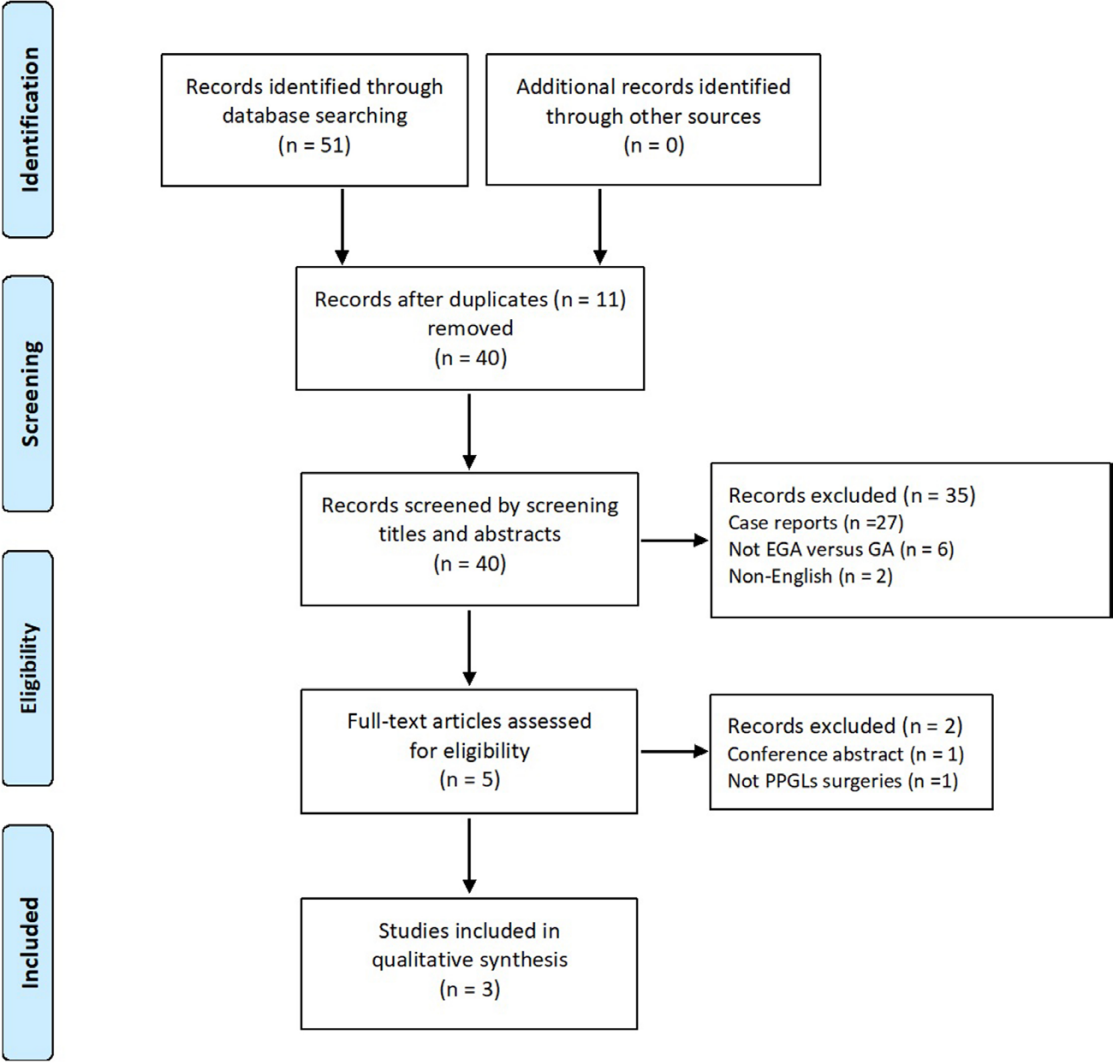
The flow diagram of study selection for this review.

A total of 51 studies were initially identified, of which 11 studies were duplications and 37 studies were excluded by reviewing the title, abstract and full-text. Finally, 3 retrospective cohort studies^[16–18]^ involving 347 patients were included (Fig. 1); among them 222 patients received combined epidural-general anesthesia and 125 patients received general anesthesia alone.

The characteristics of included studies were shown in Table 1. These 3 studies were published from 2018 to 2020, performed in China,^[18]^ Korea,^[16]^ and America,^[17]^ respectively. The surgery types of the included studies were open surgery of pheochromocytoma,^[18]^ open surgery of pheochromocytoma or paraganglioma,^[17]^ and laparoscopic or open surgery of pheochromocytoma or paraganglioma,^[16]^ respectively. In the study of Li et al,^[18]^ the general anesthesia was induced with propofol, sufentanil or fentanyl, and rocuronium. The anesthesia was maintained with sevoflurane, nitric oxide, sufentanil or remifentanil, and cisatracuriu or rocuronium. In the study of Jeon et al,^[16]^ the induction of general anesthesia was conducted with thiopental sodium/propofol/ etomidate and succinylcholine/cisatracurium/rocuronium. The anesthesia was maintained using sevoflurane/isoflurane/desflurane or propofol, along with intermittent injections of cisatracurium/vecuronium/rocuronium. Wiseman et al^[17]^ did not report the details information of general anesthesia. Li et al^[18]^ defined the primary outcome as the incidence of postoperative complications during the hospital stay. Postoperative complication was defined as newly onset conditions that reached grade 2 or higher using the Clavien-Dindo classification.^[28]^ In the study of Wiseman et al,^[17]^ the primary endpoint was postoperative hypotension which was defined as a mean arterial pressure ≤ 60 mm Hg via invasive blood pressure monitoring. Jeon et al^[16]^ focused on the intraoperative hypotension and hypertensive crisis. Intraoperative hypotension was defined as a mean arterial pressure < 60 mm Hg or a decrease > 30% of systolic intra-arterial blood pressure (SBP) baseline after ligation of the adrenal vein. Hypertensive crisis was defined as a SBP > 200 mm Hg or an increase > 30% of SBP baseline during the operation.

**Table 1:** Study characteristics.

Study	Country	Study design	Patients (number)	Age (yrs, mean ± SD)	Type of surgery	Epidural anesthesia	Intraoperative vasoactive medications	Primary outcome
Jeon 2020	Korea	Retrospective cohort study	119	48.8 ± 13.0 vs 49.9 ± 14.8	Open or laparoscopic resection of pheochromocytoma and paraganglioma	0.2% ropivacaine or 0.2% chirocaine + 3 mg of morphine sulfate or 50 mcg of fentanyl	Not reported.	Intraoperative hypotension: MBP < 60 mm Hg or SBP reduction > 30% of SBPb; Hypertensive crisis: SBP > 200 mm Hg or an increase > 30% of SBPb.
Wiseman 2020	USA	Retrospective cohort study	97	38.1 ± 15.2 vs 39.5 ± 14.1	Open resection of pheochromocytoma and paraganglioma	0.0625 - 0.5% bupivacaine with or without 0.05 mg/mL fentanyl	Not reported.	Postoperative hypotension (MAP ≤ 60 mm Hg);
Li 2018	China	Retrospective cohort study	146	42 ± 14 vs 52 ± 14	Open resection of pheochromocytoma	1% lidocaine or 0.5% ropivacaine	Antihypertensive drugs: phentolamine, urapidil, nicardipine and esmolol; vasopressors: ephedrine, phenylephrine, norepinephrine, epinephrine and dopamine.	Postoperative complications: newly onset medical conditions that required therapeutic intervention, i.e., grade 2 or higher according to the Clavien-Dindo classification.

MAP = mean arterial pressure, MBP = mean blood pressure, SBP = systolic blood pressure, SBPb = SBP immediately before induction of anesthesia.

**Figure 2.**
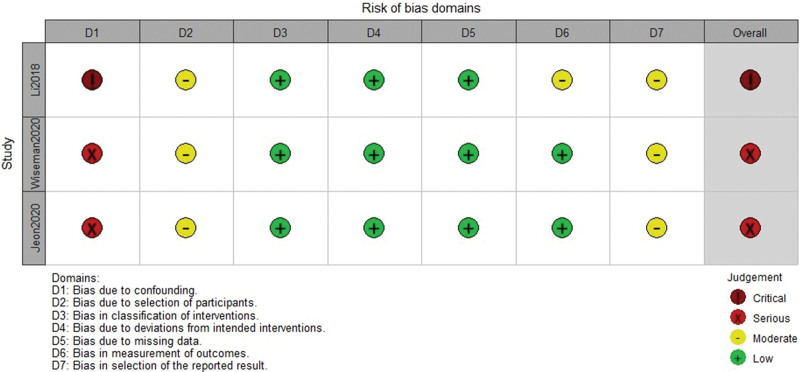
The risk of bias assessment of each domain and the overall grading of risk for each study by the Robins-I tool which contains 7 items. The estimated risk of bias for each item was rated as “low,” “moderate,” “serious,” or “critical.”

All included studies were rated as having a moderate risk of bias with the ROBINS-I tool. The details of risk of bias for each included study was present in Figure 2.

Data in these included studies were not available to be combined for meta-analysis as each predefined outcome was reported in only 1 study. Li et al^[18]^ reported the incidence of postoperative complications in patients undergone open surgery of pheochromocytoma. They found that the incidence of postoperative complications was lower in patients with combined epidural-general anesthesia than in those with general anesthesia alone (6.0% [6/100] vs 23.9% [11/46], *P* = .006). Furthermore, the severity of postoperative complications was lower in patients with combined epidural-general anesthesia compared with general anesthesia alone (*P* = .017). Additionally, combined epidural-general anesthesia was identified as one independent factor (Odds ratio [OR], 0.219; 95% confidence interval [CI], 0.065–0.741; *P* = .015) that associated with lower occurrence of postoperative complications using multivariate Logistic regression.

Wiseman et al^[17]^ compared the effects of combined epidural-general anesthesia with general anesthesia alone on the incidence of postoperative hypotension in patients undergone open resection of PPGLs. They showed that patients with epidural anesthesia had a higher incidence of postoperative hypotensive compared to those without epidural anesthesia (58.8% [40/68] vs 25.0% [7/28], *P* = .003). Moreover, epidural anesthesia was demonstrated as an independent factor associated with postoperative hypotension (Hazard ratio, 3.49; 95% CI, 1.25–9.76; *P* = .017).

Jeon et al^[16]^ compared the effects of combined epidural-general anesthesia with general anesthesia alone on hemodynamic instability in patients during PPGLs surgeries. The incidence of intraoperative hypotension was higher in patients with combined epidural-general anesthesia than in those with general anesthesia alone (88.7% [47/53] vs 64.7% [33/51], *P* = .008). No difference was found in the incidence of hypertensive crisis (50.9% [27/53] vs 58.8% [30/51], *P* = .542). The combined epidural-general anesthesia was suggested to be an independent predictor of intraoperative hypotension (Adjusted OR, 5.439; 95% CI, 1.410–20.977; *P* = .014), but had no association with hypertensive crisis (Adjusted OR, 0.378; 95% CI, 0.141–1.015; *P* = .053).

## 4. Discussion

During surgical resection of catecholamine-secreting tumors, mainly including pheochromocytomas and paragangliomas, hemodynamic instability can occur and be life-threatening.^[29,30]^ Epidural anesthesia has been reported beneficial to the hemodynamic fluctuations and the postoperative complications associated with PPGLs surgeries.^[18,22]^ Although epidural anesthesia has been commonly used as a supplementary technique of general anesthesia in PPGLs surgeries, it is unclear whether combined epidural-general anesthesia was better than general anesthesia alone for these patients. Therefore, we performed a systematic review and meta-analysis, and finally identified 3 retrospective cohort studies^[16–18]^ that compared the effects of combined epidural-general anesthesia with general anesthesia alone on the hemodynamic fluctuations and postoperative outcomes in patients undergone PPGLs resections. Unexpectedly, the combined epidural-general anesthesia was suggested as an independent predictor of both intraoperative hypotension and postoperative hypotension, although it could reduce the incidence of postoperative complications compared with general anesthesia alone.

Previous studies reported that the incidence of hypotension associated with epidural block for open abdominal surgeries was about 15% to 33%,^[31,32]^ which is likely due to a decreased peripheral vascular tone.^[31]^ For PPGLs surgeries, the incidence of intraoperative hypotension was as high as 41% to 81%.^[33,34]^ One recent study demonstrated that, compared with general anesthesia alone, combined epidural-general anesthesia was associated with a higher incidence of intraoperative hypotension in video-assisted thoracoscopic lung cancer resections.^[35]^ However, there is no consensus whether epidural anesthesia leads to the intraoperative and/or postoperative hypotension in patients with PPGLs surgeries. The included studies in our review showed that epidural anesthesia was an independent risk factor for both intraoperative and postoperative hypotension of patients undergone resection of PPGLs, and the incidence of hypotension in patients with epidural anesthesia was 88.7%^[16]^ and 58.8%,^[17]^ respectively. This high incidence was also suggested to be partly caused by the abrupt reduction in circulating catecholamines after resection of PPGLs as well as by preoperative alpha/beta-blockade use.^[36]^ Kang et al compared the effects of combined epidural-general anesthesia with general anesthesia alone on hemodynamic instability during resection of PPGLs.^[37]^ The primary outcome was the occurrence rate of post-resection hypotension. Consistent with the results of Wiseman et al,^[17]^ their published conference abstract showed that combined epidural-general anesthesia was an independent risk factor of post-resection hypotension after PPGLs surgeries.^[37]^

Patients with PPGLs usually suffered from hypertension.^[38,39]^ It is necessary to control patients’ blood pressure at a desirable level prior to PPGLs surgeries.^[7]^ Uncontrolled preoperative hypertension may increase the risk of perioperative complications in patients undergoing PPGLs surgeries,^[40]^ and usually cause intraoperative hypotension due to the impaired regulation ability of blood pressure.^[41,42]^ One of the included studies^[16]^ found that a high baseline mean blood pressure (MBP) was associated with the occurrence of intraoperative hypotension during PPGLs surgeries. In their study, the cutoff value of mean blood pressure to predict intraoperative hypotension was 93.7 mm Hg.^[16]^ Similarly, 1 published abstract of Kang et al showed that increased MBP baseline was an independent risk factor of post-resection hypotension for PPGLs surgeries.^[37]^ Therefore, the baseline level of MBP may serve as a predictor for intraoperative and/or post-resection hypotension for PPGLs surgeries.

In addition of epidural anesthesia, the use of beta-blockers was suggested another independent factor that associated with postoperative hypotension for resection of PPGLs.^[17]^ Beta-blockers are usually used to control tachyarrhythmias resulting from the use of alpha-blockers in the preoperative management of patients with PPGLs.^[7,43]^ Generally, alpha-blockers but not beta-blockers are thought to be the major medication factor that leading to postoperative hypotension after PPGLs resection. Surprisingly, Wiseman et al reported that alpha-adrenoceptor blockers were not a predictor of postoperative hypotension.^[17]^ Interestingly, 1 abstract also reported that alpha-blocker premedication was not associated with post-resection hypotension in PPGLs surgeries.^[37]^ Similarly, a meta-analysis showed that preoperative alpha-blockade use had no effects on hemodynamic fluctuations compared to no alpha-blockade use in phaeochromocytoma surgery.^[44]^ Wiseman et al first identified beta-blockers as an independent risk factor of postoperative hypotension for resection of PPGLs.^[17]^ Notably, beta-blocker itself can also reduce blood pressure or exacerbate the hypotension induced by alpha-blockers.^[13,45]^ The effects on blood pressure of beta-blockers may be prominent in patients with alpha-blockers premedication for PPGLs surgeries, particularly for those received epidural anesthesia. Therefore, it should be cautious when performing epidural anesthesia on the patients with alpha-blockers and beta-blockers premedication for resection of PPGLs.

The hypertensive crisis is another common feature of hemodynamic instability with an occurrence rate of 51% to 85% in PPGL surgeries, which is usually caused by excessive release of catecholamine.^[46,47]^ Tumor manipulation is the most important factor to cause hypertensive crisis by inducing a catecholamine surge during PPGLs surgery.^[47,48]^ The combined epidural-general anesthesia technique has been reported to facilitate hemodynamic stability before tumor isolation and resection.^[18,23]^ However, one of our included studies^[16]^ showed that combined epidural-general anesthesia had no interaction with hypertensive crisis during PPGLs surgeries but was associated with hypotension. This may be explained by that the epidural block was not sufficient to suppress the catecholamine surge during tumor manipulation but exaggerated the sympathectomy and vasodilatory effects after tumor ligation.^[49,50]^

Mean attenuation (Hounsfield unit: HU) value on unenhanced computed tomography was suggested as a diagnostic tool for PPGLs.^[51,52]^ Interestingly, an increased attenuation number on unenhanced computed tomography was demonstrated as the only independent predictor of hypertensive crisis.^[16]^ Meanwhile, an increased attenuation number on unenhanced computed tomography was also an independent predictor for intraoperative hypotension.^[16]^ The underlying mechanism of this correlation remains unclear, further studies are needed to determine this.

Mounting evidence has demonstrated that epidural blockade could improve the postoperative complications of various type of surgeries.^[53,54]^ A meta-analysis showed that epidural analgesia was associated with a lower incidence of pulmonary complications after abdominal and thoracic surgeries, probably due to reduced opioid consumption, earlier mobilization, and improved cough.^[15]^ Epidural anesthesia may also contribute to improve the balance between myocardial oxygen consumption and supply^[55]^ as well as to relieve the activation of postoperative neuroendocrine, metabolic and inflammatory response.^[56]^ One of the included studies^[18]^ first reported that combined epidural-general anesthesia was associated with a lower incidence of postoperative complications in patients undergone open surgery of pheochromocytoma. However, 1 major limitation of their study was that patients’ data were collected until hospital discharge. Therefore, their results might have underestimated the incidence of postoperative complications because a prospective study^[57]^ found that about 1 to 3rd of complications occurred between discharge and postoperative 30 days. The effects of combined epidural-general anesthesia on the occurrence of overall, including both short-term and long-term postoperative complications for PPGLs surgeries need to be further assessed.

The operative approach for PPGLs resection may influence the outcomes of patients. Currently, the operation approach for PPGLs resection mainly includes laparoscopic and open resection. Patients with larger malignant PPGLs were more likely to receive open approach, while laparoscopic approach appears to be safe for tumor size < 6 cm.^[58]^ However, no difference was found in short- or long-term postoperative outcomes between these two operative approaches in PPGLs surgeries, although open approach was associated with longer duration of hospital stay.^[58]^ Likewise, the operative approach may also influence the effects of epidural block on the outcomes of patients underwent PPGLs surgeries. Among the included studies in this review, 2 studies enrolled patients receiving open surgery,^[17,18]^ while 1 enrolled patients receiving laparoscopic or open surgery.^[16]^ However, no published studies have reported the effects of different operation approach on the effects of epidural block for PPGLs surgeries. Further prospective RCTs are needed to determine this.

For adrenalectomy, several studies found that laparoscopic adrenalectomy (LA) has greater advantages than open adrenalectomy (OA) in terms of postoperative pain, patient satisfaction, and the length of hospital stay and recovery times.^[59,60]^ Moreover, LA is associated with a lower perioperative morbidity and shorter length of stay than OA.^[61,62]^ In recent years, robotic-assisted adrenalectomy is used increasingly, and a meta-analysis showed that robotic-assisted adrenalectomy is a superior technique to conventional LA in treating adrenal tumors, even in pheochromocytoma.^[63]^ Therefore, it will also be interesting to evaluate the effects of epidural anesthesia for patients with robotic-assisted resection of PPGLs.

From this systematic review and meta-analysis, we summarized several factors that may influence the outcomes regarding the use of epidural anesthesia in PPGLs, including patients gender, perioperative blood transfusion,^[18]^ the use of beta-blockers,^[17]^ the baseline of mean blood pressure, and the attenuation number on unenhanced computed tomography.^[16]^ Other factors, such us the surgical approach may also be involved in the effects of epidural anesthesia during PPGLs.^[19]^ These factors should be noted and validated in future studies when investigating the effects of epidural anesthesia in PPGLs.

There were several limitations in this study. Firstly, all included researches were retrospective cohort studies, and the number of included studies and sample were small. Only 3 studies were included in this study, the sample was 119,^[16]^ 97,^[17]^ and 146,^[18]^ respectively. Secondly, the results between included studies were not available to combined for meta-analysis due to each outcome was only reported in an individual study. Thirdly, the quality of included studies were relatively low, as the overall risk of bias were serious to critical. Therefore, large samples, multicenter, randomized controlled trials are required to determine the efficacy and safety of combined epidural-general anesthesia in surgical resection of PPGLs.

## 5. Conclusions

In summary, no published RCTs have yet assessed clinically relevant outcomes with respect to the application of epidural anesthesia during PPGLs surgeries. On the basis of the findings of non-RCT literature, the outcomes from three retrospective studies suggests that combined epidural-general anesthesia was associated with both intraoperative and postoperative hypotension, although it reduced the incidence of postoperative complications in patients undergone surgical resection of PPGLs. Therefore, in choosing between combined epidural-anesthesia and general anesthesia alone for PPGLs surgeries, potential benefits should be balanced against the risk of hypotension. Well-designed RCTs should nonetheless be encouraged to properly assess the efficacy and safety of epidural anesthesia for PPGLs surgeries.

## Author contributions

**Conceptualization:** Shuai Zhu.

**Data curation:** Min Yang, Chao Kang.

**Formal analysis:** Min Yang, Chao Kang.

**Methodology:** Min Yang, Chao Kang.

**Validation:** Min Yang, Shuai Zhu.

**Writing – original draft:** Min Yang.

**Writing – review & editing:** Shuai Zhu.
